# Supplementary figures and images for: High-Throughput Stool Metaproteomics: Method and Application to Human Specimens
Source: mSystems. 2020 Jun 30;5(3):e00200-20. doi: 10.1128/mSystems.00200-20 (PMC7329322; doi:10.1128/mSystems.00200-20)

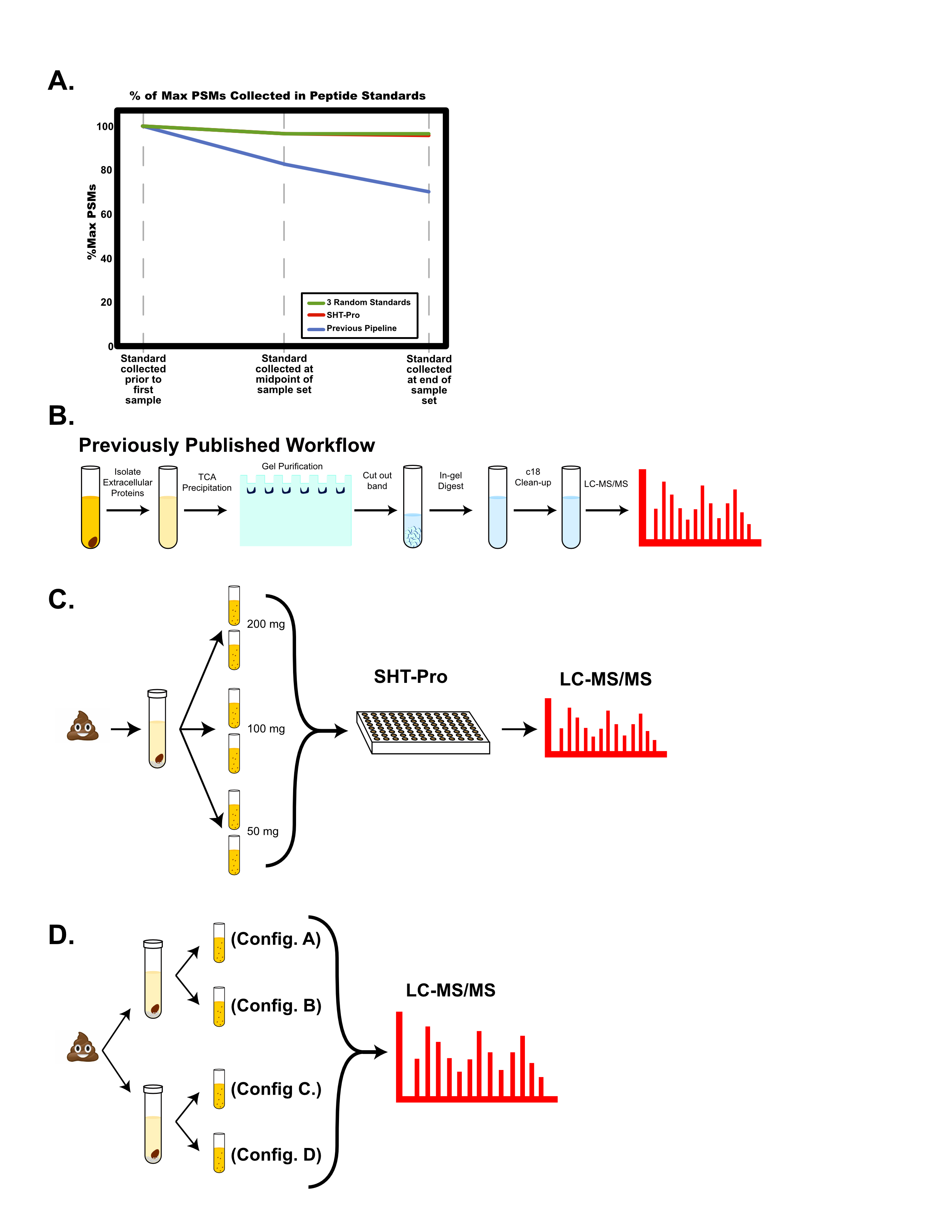

Supplement: FIG S1 [file mSystems.00200-20-sf001.jpg]

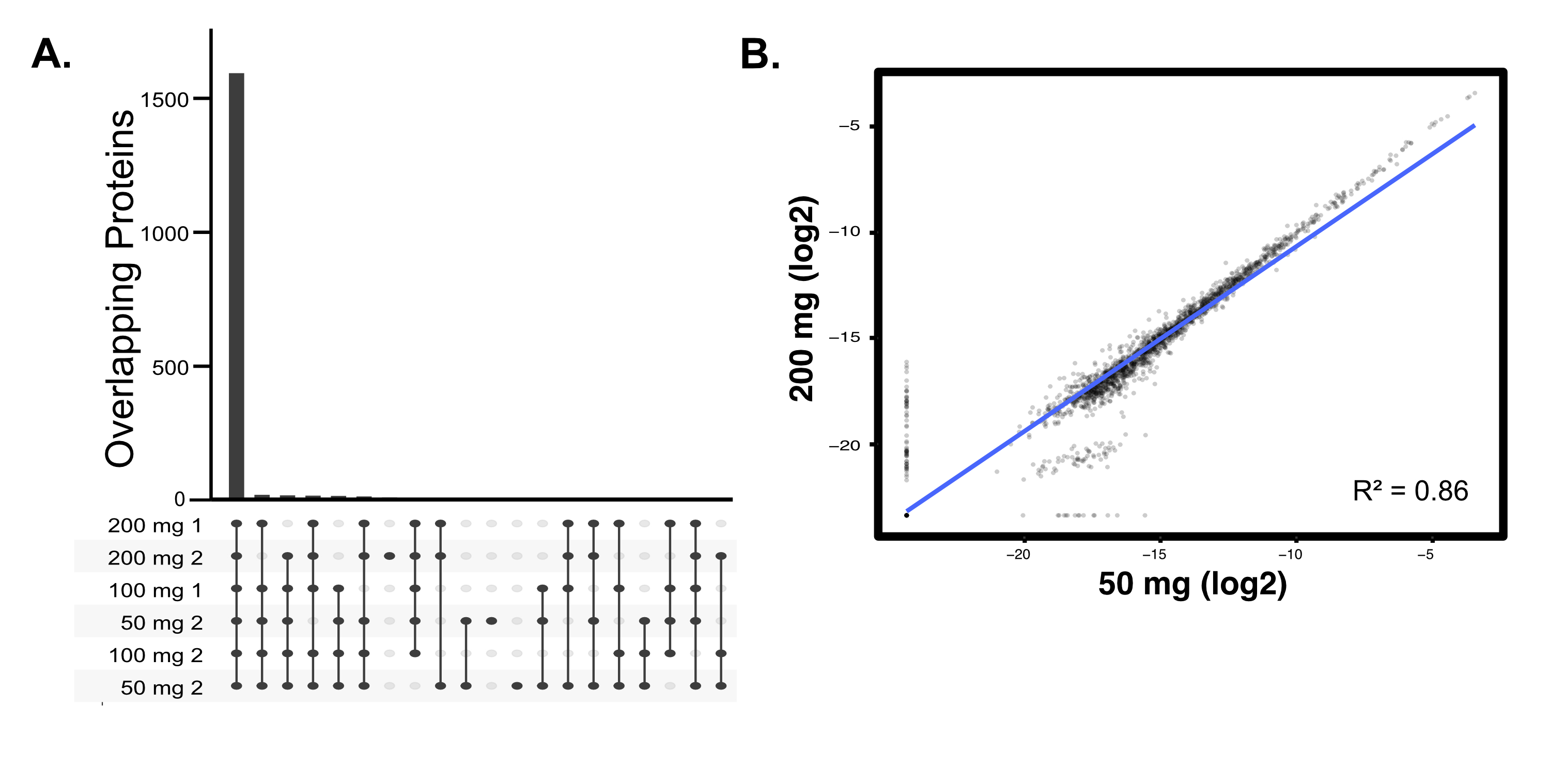

Supplement: FIG S2 [file mSystems.00200-20-sf002.jpg]

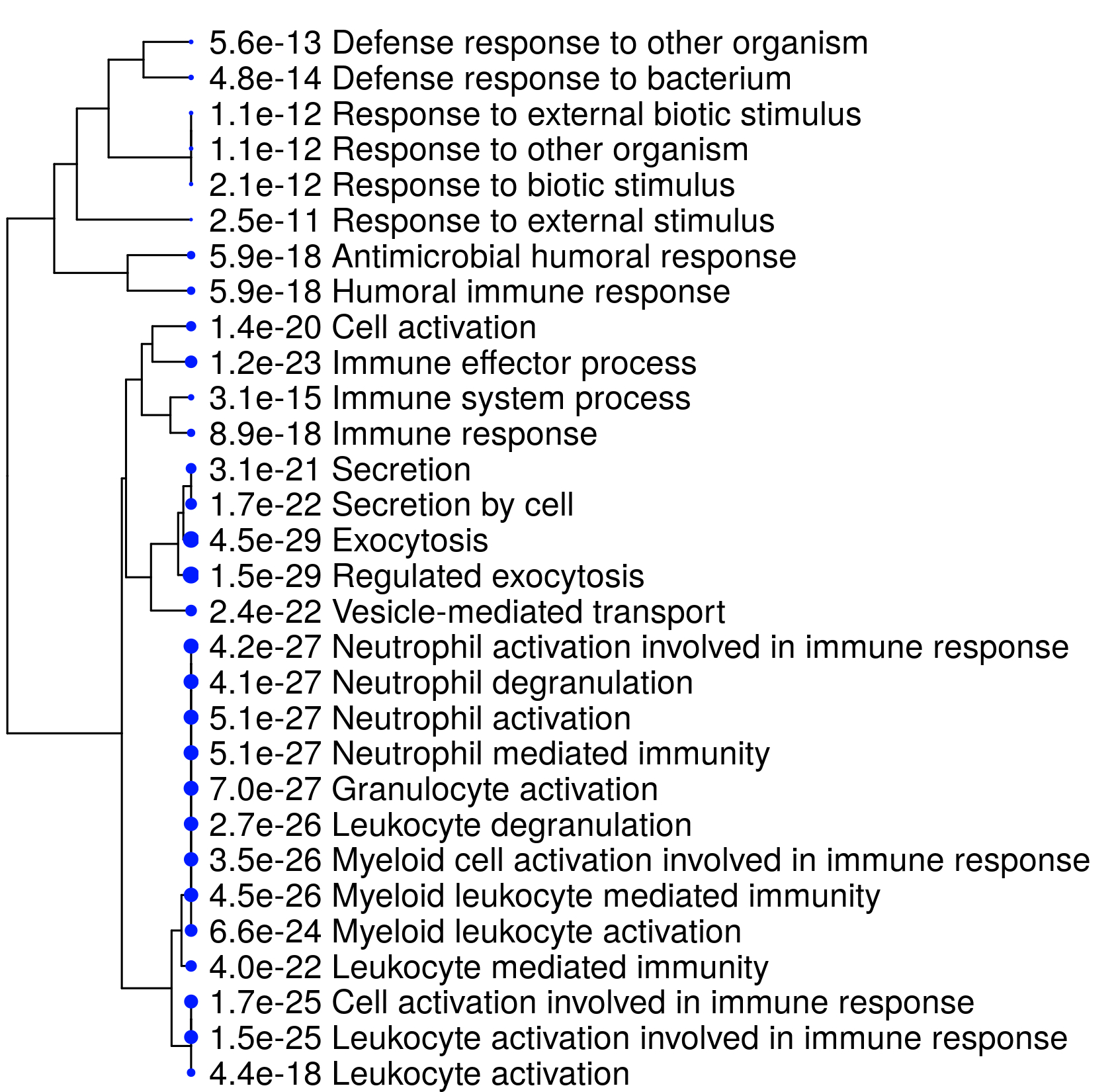

Supplement: FIG S3 [file mSystems.00200-20-sf003.jpg]

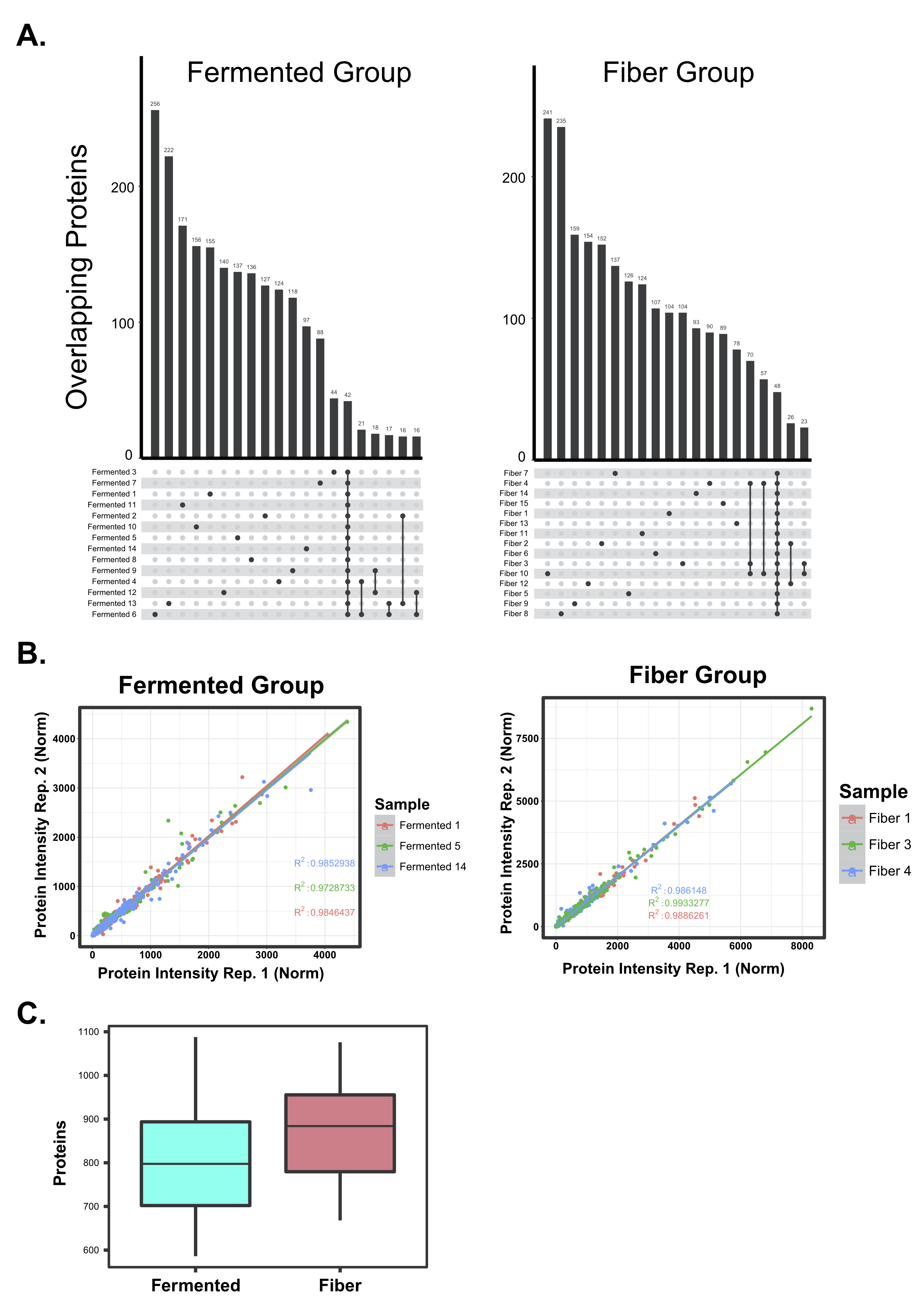

Supplement: FIG S4 [file mSystems.00200-20-sf004.jpg]

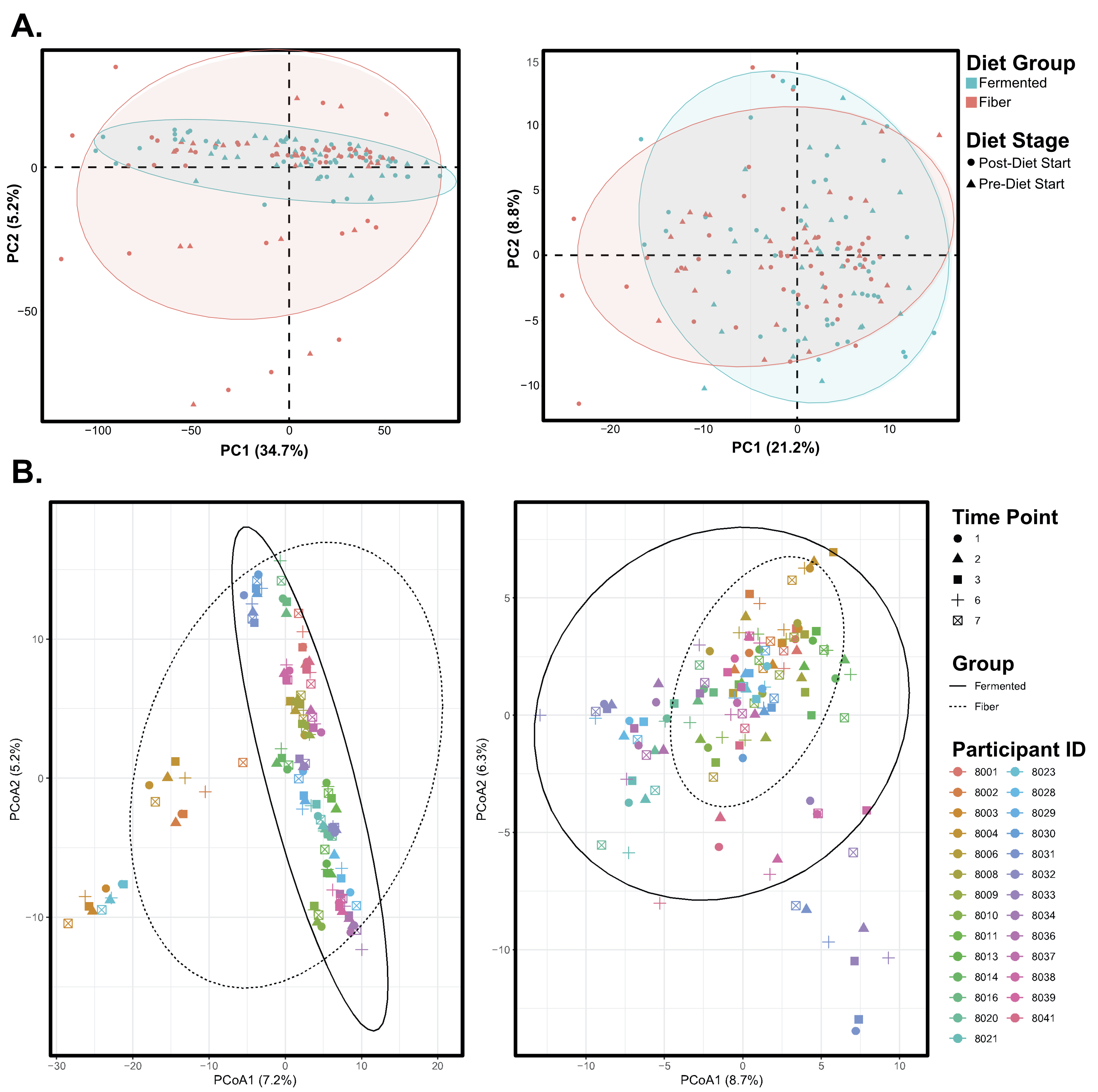

Supplement: FIG S5 [file mSystems.00200-20-sf005.jpg]

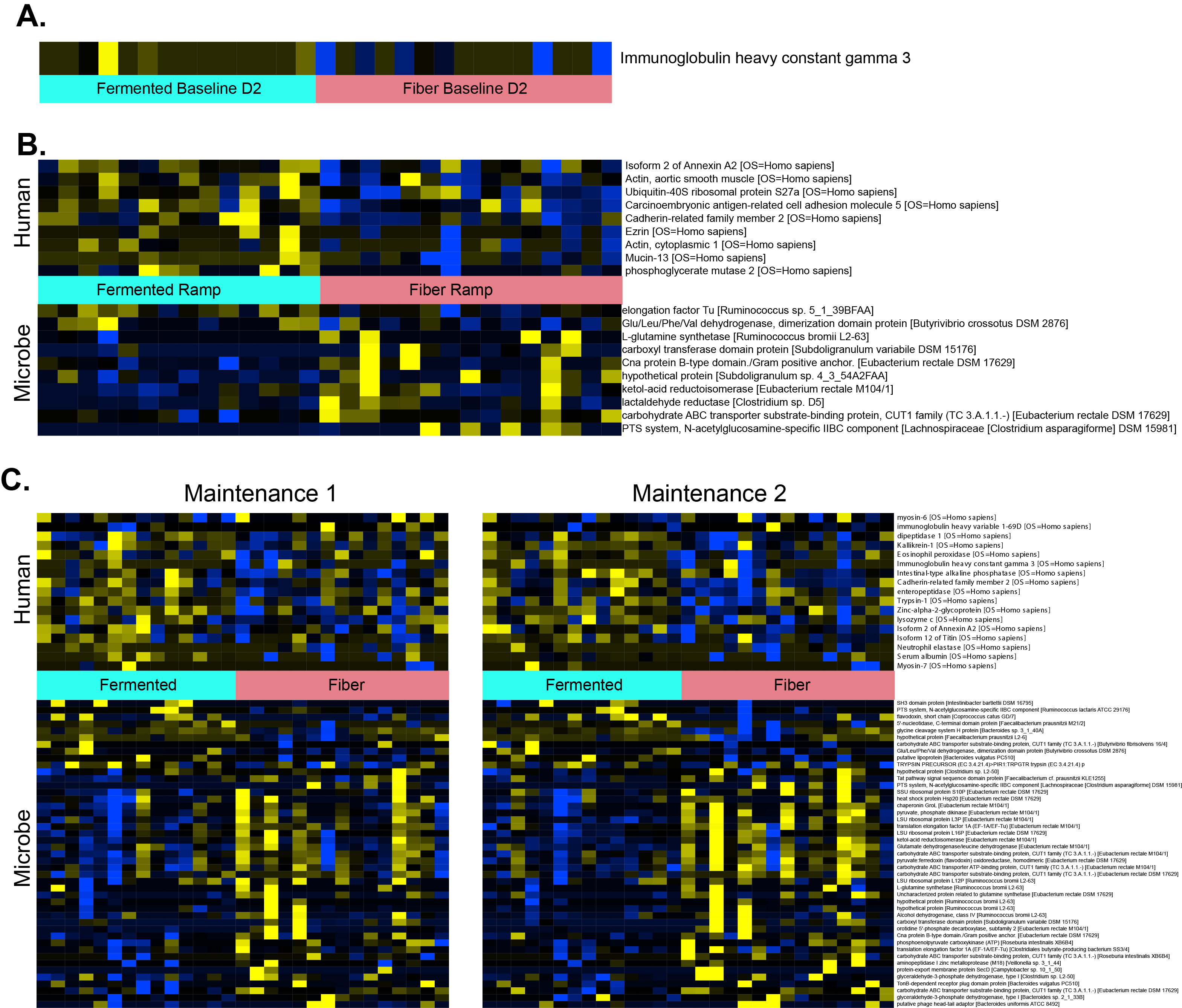

Supplement: FIG S6 [file mSystems.00200-20-sf006.jpg]
